# Wherein is the concept of disease normative? From weak normativity to value-conscious naturalism

**DOI:** 10.1007/s11019-021-10048-x

**Published:** 2021-08-30

**Authors:** M. Cristina Amoretti, Elisabetta Lalumera

**Affiliations:** 1 DAFIST, Philosophy Section, and Research Center for Philosophy of Health and Disease, University of Genoa, Via Balbi 4, 16126 Genova, Italy; 2 Department for Life Quality Studies, and Research Center for Philosophy of Health and Disease, University of Bologna, Largo Augusto 230, 47921 Rimini, Italy

**Keywords:** Disease, Harm, Naturalism, Non-epistemic values, Normativism, Value-ladenness

## Abstract

In this paper we focus on some new normativist positions and compare them with traditional ones. In so doing, we claim that if normative judgments are involved in determining whether a condition is a disease only in the sense identified by new normativisms, then disease is normative only in a weak sense, which must be distinguished from the strong sense advocated by traditional normativisms. Specifically, we argue that weak and strong normativity are different to the point that one ‘normativist’ label ceases to be appropriate for the whole range of positions. If values and norms are not explicit components of the concept of disease, but only intervene in other explanatory roles, then the concept of disease is no more value-laden than many other scientific concepts, or even *any* other scientific concept. We call the newly identified position “value-conscious naturalism” about disease, and point to some of its theoretical and practical advantages.

## Introduction

From the 1970s onwards, a major discussion in the philosophy of medicine and psychiatry has been aimed at providing an account of the general concepts of disease and mental disorder in order to discriminate them from non-disease and non-disorder conditions, such as normal biological variations, social deviances, or moral failings. In this debate, conditions that might be ordinarily distinguished from diseases and mental disorders, such as illnesses, wounds, or injuries, are all lumped together, as the main goal of the discussion is to differentiate pathological and non-pathological conditions. In what follows, then, we will use ‘disease’ as an umbrella term that refers to all pathological conditions, both somatic and mental. In trying to define the general concept of disease scholars were soon divided between normativists and naturalists, with normativists claiming that norms and values are necessary in order to define what a disease is (section Some ‘traditional’ normative positions), and naturalists denying it, and defending disease accounts in terms of biological dysfunction^1^

While some philosophers recommend that the attempt to define the general concept of disease should be abandoned altogether due to its lack of progress (Ereshefsky, 2009; Hesslow, 1993; Kincaid, 2008), others propose alternative (Broadbent, 2019; Khushf, 2007),^2^ or more nuanced ways of framing it (Cooper, 2020; Kingma, 2014). Some attempts acknowledge for instance that even if the concept of disease does not have a straightforward normative analysis, values still have a role in demarcating diseases from non-diseases. Elseljin Kingma (2014) pointed out that ‘value intrusion’ can be found in the operationalization of the notion of function and/or in the justification of such an operationalization, thus introducing the idea of a ‘third-level’ and a ‘fourth-level’ normativism (section Kingma’s ‘third- and fourth-level’ strategy). More recently, Rachel Cooper (2020) argued that, in spite of a shift of the concept of disorder towards naturalism, normativism can still be defended with a “belt and brace” strategy, that is, arguing that values are needed to fix the threshold between pathological conditions and non-pathological conditions of low or high functioning, as well as in demarcating between diseases and unfavorable environmental conditions (section Cooper’s “belt-and-brace” strategy).

In this paper, we claim that if normative judgments are involved in determining whether a condition is a disease only in the sense identified by Kingma’s ‘third- and fourth-level’ or Cooper’s ‘belt-and-brace’ strategies, then the concept of disease is normative only in a weak sense. In fact, such a *weak normativism* characterized by ‘value intrusion’ can be better explained in terms of interest-ladenness or contextual dependence, that is, in terms of properties that many other scientific concepts may easily have without being tagged as fully normative. However, weak normativism is very different, both in motivations and consequences, from *strong normativism*, which is advocated by traditional normativists about disease, who claim that disability, action failure, harm, suffering, unluckiness, undesirability, and other value-laden concepts that imply a negative evaluation are explicit conceptual components of the concept of disease. Specifically, we shall argue that weak and strong normativism are different to the point that one ‘normativist’ label ceases to be appropriate for the whole range of positions. So, the focus of our discussion is that weak normativism is compatible with, and is a possible complement to, naturalism about disease. It is worth noting that in this paper we will neither argue against normativism, either weak or strong, nor will we defend naturalism by claiming that Kingma’s and Cooper’s strategies fail. This is why we can express our main claim with a conditional: *if* values and norms are not explicit components of the concept of disease, but only intervene in other explanatory roles, *then* the concept of disease is no more value-laden than many other scientific concepts, or even *any* other scientific concept.

On the theoretical side, the point we make is relevant to the demarcation problem, which is arguably one of the reasons why the normativist-naturalist debate is still alive. On the practical side, our argument clarifies that the concept of disease reflects the fact that medicine, as an applied science and profession, is embedded in a larger cultural and social environment, where ethical and prudential reasons may intervene when purely epistemic reasons fall short of settling an issue.

The structure of the paper is the following. In section Some ‘traditional’ normative positions, we will set the stage by presenting what we take to be the most representative normative positions in the debate on the concept of disease, that is, those advocated by Nordenfelt (1986, 1987), Fulford (1989), Megone (1998, 2000), Reznek (1987), Cooper (2002), and Wakefield (1992). Kingma’s ‘third- and fourth-level’ and Cooper’s ‘belt-and-brace’ strategies will be discussed in detail in sections Kingma’s ‘third- and fourth-level’ strategy and Cooper’s “belt-and-brace” strategy respectively, after having explained the different philosophical projects to which they belong. In the light of this, in section Weak and strong normativity we will introduce our distinction between *weak* and *strong normativism* and in section Weak/strong normativity vis à vis with other similar distinctions we will illustrate how our position differs from those of Boorse (1975), Khushf (2007), Kingma (2014), and Cooper (2020), who have all mapped the different varieties of normativism. In section Towards a better framework for the normativist-naturalist debate about disease our discussion will be wrapped up, and we will advance a proposal on how the naturalist-normative debate may go on—to anticipate, towards a value-conscious naturalism, with different values involved, in different areas of clinical medicine and healthcare, in determining which conditions should be treated and how.

### Some ‘traditional’ normative positions

In this section, we will briefly sum up in what sense some prominent normative theories of disease are indeed ‘normative’. In so doing, we are not aiming at fully describing or explaining the various positions, but rather at highlighting what they have in common and what makes all of them ‘normative’ in a traditional and widely recognized sense. As we will show, traditional normativists typically consider the concept of disease holistic and evaluative: on the one hand, it regards the whole person, not single parts of an organism; on the other hand, disease is judged a bad thing to have, something that we (individuals or society) negatively evaluate and dislike it being constitutively linked to some disability, action failure, harm, suffering, unluckiness, or undesirability. Typically, traditional normativists deny that disease can be analyzed (solely) in terms of dysfunction and underlie the necessity of including evaluative terms as its explicit conceptual components. In what follows, we will address the positions held by Nordenfelt (1986, 1987), Fulford (1989), Megone (1998, 2000), Reznek (1987), Cooper (2002), and Wakefield (1992)^3^.

An influential set of normativist theories, sometimes dubbed under the label of ‘embedded instrumentalism’ (Richman & Budson, 2000), relate the concept of disease with those of disability and action failure (Pörn, 1977; 1984; Whitbeck, 1978, 1981). More precisely, instrumentalists consider the concept of health, not that of disease, to be primary: they first try to determine what health is and then to infer what the notion of disease amounts to. In very general terms, health is considered a good thing to have as being healthy means having the ability to perform the appropriate actions required to achieve certain goals. Derivatively, disease is considered a bad thing to have as being diseased corresponds to the lack of this ability; the concept of disease is thus characterized in terms of disability and judged secondary to that of health. In this way, the characterization of the concepts of health and disease can obviously change depending on how abilities and goals are defined but it always maintains a holistic and evaluative dimension. On the one hand, health and disease refer to the whole person, considered in their biological, psychological, and social dimension, while, on the other hand, they must be assessed with regard to one’s own goals and values, which are non-factual components. Among embedded instrumentalisms, Lennart Nordenfelt’s welfare theory of health (or holistic theory of health) is one of the most prominent (Nordenfelt, 1986, 1987). Roughly, it is based on the intuition that to be healthy is to have the ability^4^—given standard circumstances—to achieve one’s most important goals, or one’s all ‘vital goals’, that is “those goals which are necessary and jointly sufficient for [one’s] minimal happiness” (Nordenfelt, 1987, p. 79). As health is the primary concept, illness can be derivatively characterized as any deficiency in health, while disease as a type of internal condition most instances of which cause illness. Specifically, diseases are internal conditions that jeopardize one’s ability to achieve one’s own vital goals and, thus, a minimal degree of happiness. In this sense, the notion of disease is not only holistic, as vital goals and health must be determined with regard to the person as a whole, but also evaluative from the very beginning, as a certain condition would count as a disease only if it is related with the experiential notions of illness, action failure, and dislike, and thus if it is judged in a negative way, as a bad thing to have. To sum up, according to Nordenfelt’s theory, disability, vital goals, and happiness—all notions that are intrinsically value-laden—are conceptual components of the notion of disease and are thus necessary to define what a disease is.

The second normativist position that we briefly address is that of Bill Fulford (1989) who starts from an analysis of how medical concepts (disease, illness, malady, etc.) are characterized in ordinary language. To begin, Fulford explicitly endorses an ‘ethics-based’ account of medical concepts, as he notes that in ordinary language their meanings are all largely linked to a negative value judgment. Notably, he maintains that the primary concept in medicine is not disease but illness, that is “the patient’s direct experience of something wrong” (1989, p. 262), and that the concept of illness is governed by evaluative rather than factual elements. Specifically, the negative experience of something wrong characterizing illness is of a distinctive medical kind which involves action failure, that is, “a failure to do something which is *not* inherently difficult, a failure to do something which one would ordinarily just get on and do” (1989, p. 118), such as lifting up an arm, despite the apparent absence of oppositions and/or impediments; in this perspective, illness can also refer to unpleasant sensations from which one fails in the effort to withdraw. So, illnesses include conditions that interfere with normal actions or unpleasant sensations that cannot be blocked by normal actions. According to Fulford’s theory, the notion of disease is parasitic to that of illness in that all diseases are linked in some way to the production of illness: diseases are conditions that are largely considered to be illnesses, or tend to produce illnesses, or are causally related to illnesses. As illness is linked to action failure, disease is also linked to action failure; it is thus clear that the notion of action failure is a conceptual component of that of disease. In this sense, disease is not only holistic, as illnesses and action failures involve the whole person, but also intrinsically evaluative, as illness and action failure are negative value terms, not factual ones.

Nordenfelt and Fulford—though in different ways—both claim that a disease is a bad thing to have and negatively evaluate it because it involves disability and action failure. Other normativists, however, consider a disease a bad thing to have and negatively evaluate it because it causes some harm and suffering, that is, it hampers or inhibits human flourishing and well-being. For instance, Chris Megone (1998, 2000), following Aristotle, argued that a disease is whatever threatens flourishing human life. In the case of humans, Megone claims that flourishing human life cannot be identified with mere survival and reproduction but must rather coincide with rational life, where ‘living rationally’ means not only acting in an intelligent way, but also behaving morally, following the right values and desires. If a disease is a failure to realize rational life, then the notion of disease is not purely factual but essentially evaluative, as values and goals are necessary to identify what a rational life is. Moreover, disease is also a holistic notion, as it can only be determined with regard to the whole person.

In a more elaborate account—which still focuses on the hampering of human flourishing—Lawrie Reznek (1987) argued that a certain bodily or mental condition amounts to a disease if and only if it is abnormal, requires medical intervention (that is, medical intervention is both necessary and appropriate), is not voluntary, and harms standard members of a species in standard circumstances. Focusing on the notion of harm, Reznek identifies it with whatever makes a subject less suitable for achieving his or her own good, that is, a worthwhile life—which for human beings is equivalent to the possibility of satisfying deserving desires and pleasures. It is thus clear that the concept of disease is not based on purely factual elements but is rather intrinsically holistic and evaluative: it contains the notion of harm as a conceptual component and such a notion—being dependent on those of good and worthwhile life—can only be determined with regard to the values and goals that standard individuals, considered as whole persons, would have in standard situations. Similarly, Cooper (2002) claimed that the concept of disease aims to pick out those conditions that, through being harmful, are of interest to us as people. Specifically, she identifies disease with a condition that is not only a bad thing to have, but also it is such that we would consider the afflicted person to be unlucky, and it can potentially be medically treatable. To paraphrase the first requirement, we may rather say that a bad thing to have is something harmful; contrary to Reznek, however, Cooper claims that a disease must be harmful to the individual subject, not to standard human beings or society. This means that, in principle, one and the same condition can be harmful for one subject but not harmful for another, depending on individual values and goals. Moreover, being unlucky can be regarded as a non-purely factual component too, as it means that one could reasonably have expected to be better off—that is, in the closest counterfactual worlds to the real world one would have been in a preferable state—where such an evaluation cannot ignore values and goals^5^. So, harm and unluckiness would be evaluative conceptual components of the general concept of disease, and they make it both holistic and value-laden.

The notion of harm can be a conceptual component of hybrid accounts of disease, too, such as that famously defended by Jerome Wakefield (1992), who identifies a disease with a harmful dysfunction. Putting aside the notion of dysfunction, Wakefield believes that the notion of harm is intrinsically holistic and value-laden: a harmful condition is a condition that, under current environmental circumstances, impinges in a significantly damaging way on the subject’s overall well-being, where such well-being must be assessed in relation to social standards, values, and meanings. In other words, the notion of harm depends on a negative evaluation according to some social or cultural norms and is thus linked to the social (normative) world to which the subject belongs, not to the natural (descriptive) world.

To sum up, different as they might be, both in details and motivations, all the above positions about the disease concept share some common features that make them ‘normative’ in a traditional and widely recognized sense: on the one hand, disease is seen as a holistic concept, as it refers to the whole person; on the other hand, it is constitutively evaluative, as it does not (solely) depend on factual elements but has values and norms as a necessary conceptual component: a disease is negatively evaluated and disliked as it essentially involves disability, action failure, harm, suffering, unluckiness, or undesirability. To put it another way, all the above positions can be captured by the label ‘value-requiring’ proposed by Schwartz (2007a), as they explicitly define disease using value-laden concepts.

In the next two sections we will come nearer to the focus of our discussion by examining two different ways in which non-factual elements, such as values and goals, figure not in the definition of the concept of disease as its explicit components, but rather in other explanatory roles. In what follows we will make explicit what such roles are, so let us use this vague expression for now.

### Kingma’s ‘third- and fourth-level’ strategy

Our first case study dealing with ‘value intrusion’ will be Kingma’s ‘third- and fourth-level’ strategy (Kingma, 2014). A few words about her general project are in order. We take the question behind her discussion to be similar to ours: Wherein can the concept of disease be normative? Like ours, her aim is not to take part in the debate directly but to clarify and reframe it. She proposes a methodological advance to the normativist-naturalist debate introducing four ‘levels’ or ‘four different domains where naturalist and normativist claims can be contrasted’: (1) ordinary usage or ‘practical domain’, (2) conceptual analysis, or what she calls ‘conceptually clean versions’ of health and disease, or ‘theoretical domain’, (3) the operationalization of dysfunction, and (4) the justification for that operationalization (Kingma, 2014, pp. 590–591). She takes Boorse’s biostatistical theory as the paradigmatic naturalist account, stating that “health is normal functioning, where the normality is statistical and the functions biological” (Boorse, 1977, p. 542), and “disease is only statistically species subnormal biological part-function” (Boorse, 1997, p. 4). Kingma also assumes that some version of conceptual analysis is possible and useful in philosophy of medicine. We will take both assumptions for granted in what follows.

Kingma just briefly mentions the first level, that is, the domain of ordinary language, where, she observes, the naturalist is really a straw-man. In her words “No naturalist claims that a completely value-free account of health and disease should directly drive our social decision-making, or that it corresponds exactly to how lay-people might use health and disease in a social, applied context” (Kingma 2014, 592)^6^. Then she considers the second or the theoretical level, where naturalism and normativism are traditionally opposed. As said, however, the stark opposition between naturalism and normativism in the domain of conceptual analysis is not what Kingma focuses on in her discussion—and neither will we. We then move to arguments for value-ladenness that question whether “assuming that an analysis of disease as dysfunction is correct, the concepts of function and dysfunction can themselves be defined or operationalized in value-free terms” (Kingma, 2014, p. 594). According to Kingma, this is the third level where normativism can be found, even though, she acknowledges, a few normativists have taken this route, and the discussion is more often a debate within naturalism. What is it for disease to be normative, at the third level?

‘Value-intrusion’, explains Kingma, can take place if value-free solutions to the *line-drawing problem* fail. The line-drawing problem was first introduced bySchwartz (2007b)^7^. Given that function and dysfunction of an organism, or of one of its parts, categorize a continuum of states, how are we to draw the category boundary? How are we to decide, for example, that female infertility at 20 years of age is dysfunctional, and at 70 is not? Or that anomalous prostate tissue with Gleason score 7 is cancer, for a 50-year-old man, while prostate tissue with Gleason score 5 is not cancer^8^? Boorse’s own solution is based on statistics and normal distributions of values within a reference class: if a body part or system’s level of functioning falls in the lowest percentile (bottom 5%) for people of the same sex and age, then it is dysfunctional. In Boorse’s own words, a condition is a dysfunction only if it “falls more than a certain distance below the population mean” (Boorse, 1977, p. 559). Where exactly? This can be conventionally decided, within the range of values that fall into the area. This clearly explains the infertility case just mentioned: in the reference class of 20-year-old women, those with an infertility condition are a small percentage. The cancer case is slightly less intuitive, for it involves assessing the abnormality of the cell tissue, but it can be accommodated in a similar way by Boorse’s approach.^9^ Convention is meant to solve the problem of vagueness, also known as the Sorites problem—just like when, for example, it is conventionally decided that the line between rich and poor, with respect to entitlement to a tax exemption, is drawn at 40,000 euros net income, and that 39,999-euro earners are exempted, but 40,000-euro earners are not, it can be conventionally decided how flexible the 5% percentile can be, for a given health indicator.

As Schwartz and Kingma argue, however, a further issue—in addition to, and independently of vagueness—can be raised about Boorse’s approach to the line-drawing problem. One can conceive of *unhealthy populations,* namely, reference classes where levels of functioning of an organ or system are such that a pathological outcome is very common, and of *healthy populations,* where a pathological outcome is very rare, i.e., below 1%. An example of an unhealthy population are pregnant women with respect to eclampsia (Kingma, 2014), or 80-year-old males with respect to prostate tissue lesions, while an example of a healthy population is 20-year-old females with respect to heart failure (Schwartz, 2007b). In such populations, purely statistical information would ground very counterintuitive disease judgments, for example, that eclampsia and Gleason 7 tissue lesions are not diseases, or that no one in the reference class considered has a heart failure condition.

According to Schwartz, a clause specifying the consequences that a condition brings about for the individual could solve the problem. By quantifying the negative consequences of the values of a health parameter, we can modify the cut-off point between disease and non-disease in different populations. In Kingma’s words:

if a large proportion of outputs is bad - as is the case in heart function in 75-year-old men - then the cut-off between normal and pathological shifts to a higher percentage of the population distribution: to the percentage that suffers these negative consequences. If only very few outputs are bad, such as in heart function in young women, this means that the cut-off between the healthy and the pathological shifts again to the level at which outputs start to have negative consequences, which is now a very low percentage of the population distribution (Kingma, 2014, p. 596).

Schwartz provides common sense constraints on how to conceive of negative consequences: they should encompass more than just effects on survival and reproduction, but less than anything that has negative valence to the individual. He then proposes to characterize them as “effects that significantly diminish the ability of the organism to carry out an activity that is generally standard in the species and has been for a long period of time” (Schwartz, 2007b, p. 379).

This is exactly the point where Schwartz’s solution to the line-drawing problem between function and dysfunction *can* fail, according to Kingma: if specifying negative consequences requires the notion of standard activity, then it had better be a value-free notion, in order to fit in a naturalist perspective, but is it? Can we provide a value-free account of being a *standard* activity? Though she does not aim at providing an argument for claiming that Schwartz’s solution does fail in this respect, Kingma thus individuates the third possible level of normativism as value intrusion.

Kingma’s fourth dimension of the debate between normativism and naturalism is easier to exemplify than to characterize. To say what normal functioning is, we have to determine a reference class, and in Boorse’s view, the appropriate reference class is, by default, sex and age. However, sex and age are just two out of a range of alternative parameters for reference classes—including for example socio-economic status, place of birth, and even more bizarre alternatives—and arguably the choice among the alternatives can only be made for value-laden reasons (Kingma, 2007; Stegenga, 2018, p. 24). The characterization of the fourth-level normativism is thus as follows:

Even if a definition of health or function in completely value-free terms succeeds, that definition asserts certain parameters [...] that may be describable in value-free terms, but cannot be justified in a value-free way; they might have been stated differently, and using some rather than others may be driven by, and thus reflect, a normative judgment or evaluative choice (Kingma, 2014, p. 600).

According to Kingma, this is also the case of the notion of function: even granting that an account of disease in terms of dysfunction was completely feasible, then the problem of justifying in purely naturalistic terms which account of function is adequate would remain open. In general, when goals, interests, and non-epistemic reasons are involved in the choice of one specific naturalist account over the others, then value-intrusion takes precedence, and normativism kicks in.

### Cooper’s “belt-and-brace” strategy

In this section we illustrate and discuss another recent version of the idea that there may be “value intrusion” in a concept of disease with a naturalistic core, recently elaborated by Cooper (2020). Cooper’s project is different from Kingma’s as she starts by describing a case of conceptual shift that especially occurs in psychiatry and in philosophy of psychiatry. A conceptual shift happens when a concept that describes the uses of a certain community (scientific or other) loses some features and acquires others, so that it modifies its extension. Talking about conceptual shift is therefore locating oneself within the area of descriptive conceptual analysis, which, according to Cooper (2020, p. 150), means “to make explicit our current thinking (and then potentially go on to critique it)”—referring to (Nordenfelt, 1993)^10^.

The conceptual shift Cooper concentrates on involves the concept of mental disorder^11^ endorsed by the psychological and psychiatric community, and it was made explicit by the recent decision to modify the general definition of mental disorder contained in the introduction of the fifth edition of the Diagnostic and Statistical Manual of Mental Disorder, DSM-5 (American Psychiatric Association, 2013)^12^. Specifically, in DSM-5, harm is no longer a necessary requirement for a condition being a mental disorder, but merely a “usually associated” feature that disorders may bring (Amoretti & Lalumera, 2019; Cooper, 2015b, 2020). Cooper briefly recalls that the harm criterion has reflected the social and scientific consensus since the Eighties, when homosexuality was eventually excluded from the psychiatric nosology precisely because it was admitted that it is not a harmful condition, and notices that such consensus is less firm than before. This has consequences on the discrimination problem of diseases from non-diseases, as some conditions, such as tics, can now be classified as mental disorders even in cases where they are not disadvantageous to the person who has them—whereas others, such as, paraphilic conditions, count as diseases precisely because they cause harm^13^ (Cooper, 2020, p. 148).

Cooper’s point in the paper is that, despite the shift towards naturalism, the concept of disease (disorder, in her analysis) “currently requires normative judgments to be made at multiple points. While any particular conceptual tie may give way, the whole can be expected to hold” (Cooper, 2020, p. 153). The second sentence of the quote explains why she calls her strategy in favor of normativism “belt-and-brace”. Let us see what these points are.

The first is the threshold problem, that is, the line-drawing problem that we already described in the previous section. Boorse’s biostatistical theory has it that the line between disease and non-disease is conventionally fixed somewhere around two standard deviations from the norm. However, Cooper argues, in a case like hypertension the condition that the medical community agrees to call “disease” is located at the very tail-end of a normal distribution, whereas in cases like attention deficit hyperactivity disorder (ADHD) the condition has a high prevalence, about 30%–45% in some populations. In such cases, Boorse’s strategy is not adequate to represent medical consensus. This is a version of the healthy/unhealthy population objection raised by Schwartz, and discussed by Kingma, which we illustrated above. Cooper acknowledges Schwartz’s proposal of including negative consequences in the picture, as well as Kingma’s suggestion that such a move would involve value judgments (Cooper, 2020, p. 154). Elaborating on this point, Cooper explains that for ADHD the cut-off point was fixed with the aim of individuating an impairing condition that calls for treatment, and for hypertension the cut-off point shifted as randomized controlled trials showed at what blood pressure level treatments were beneficial. The implicit last step in her argument can be made explicit as follows: since judging that a condition is impairing, calls for treatment, or instead is beneficial is grounded in practical and moral values, the solution to the line-drawing problem is value-laden and, to conclude, the concept of disease is normative.

The second point where, according to Cooper, the concept of disease requires value judgments is what she calls the *location problem*. In her words: “whether we count a problem as an internally located disorder or as an externally located environmental problem, depends on whether we think it best to attempt to ameliorate the situation by altering the individual or the environment. This depends on what types of intervention might be possible, but also on whether we think that any possible environmental accommodations are reasonable or not. Determining which environmental adjustments would be reasonable depends on a range of considerations—practical and economic, but also ethical and political” (Cooper, 2020, p. 157, see also (Cooper, 2017)). As an example, she proposes the case of a wheelchair user, who has difficulties in living well in a town without ramps but would not have any in a wheelchair-friendly environment. According to her, it is contested that the wheelchair user has a disease, and not, rather, that he or she lives in an environment that should be changed.

One may be tempted to reply that the location problem does not arise at all for a naturalist, who believes that a disease is some kind of dysfunction. In the wheelchair user’s case, the naturalist would start from the premise that diseases are dysfunction-requiring, add that the wheelchair user’s motor system is functioning below typical efficiency, and easily conclude that his or her condition is classified as a disease, whether or not the environment makes it also a disabling condition. This is the core of the relational model of disability, according to which disability is relational, whereas impairment, or disease, are located within the individual^14^. Incidentally, the same pattern of reasoning was employed by Wakefield with an anti-pathologizing agenda. According to Wakefield, requiring the presence of a dysfunction prevents one from diagnosing female primary orgasmic dysfunction to women who are just experiencing an unfavorable relational environment (Wakefield, 1988).

However, Cooper’s location problem can be made more demanding by suggesting that the naturalist rests on an implicit assumption about what is a normal or good environment for humans to live in. Kingma (2010) took this path and concluded that an identification of these environments most likely would involve judgments about norms and values, thereby spoiling value-freedom. Hausman (2012, 2014) contested the conclusion, proposing a value-free account of normal environments. Here, however, we do not need to dwell on the details of his solution. What matters to our conceptual map is that Cooper’s location problem can be recognized as a case of the problem of normal environments, and therefore it qualifies as a case of third-level normativism, as described above (Kingma 2014, p. 600). In fact, to the question ‘Wherein is disease normative?’, the location problem, just like the reference class problem, points to the following answer: in the operationalization of the notion of function.

### Weak and strong normativity

In this section we introduce what we take to be the distinction between ‘weak’ and ‘strong’ normativity with respect to the disease debate, describe weak normativity in more detail, and insist on the differences between the two notions.

In our view, the disease concept is strongly normative if an evaluative concept, such as disability, action failure, harm, suffering, unluckiness, or undesirability is one of its explicit components, or a necessary criterion for its application. For those who reject conceptual analysis, disease is strongly normative if values and goals are a central explanatory tool of a general theory of diseases. If the concept of disease is strongly normative, it is typically holistic, too, as it refers to the whole person, not to parts of an organism. We are aware that this may not be a logically perfect definition, but we believe it may convey a sufficiently precise idea. We intend the list of evaluative ingredients to be potentially open, and we offered some examples in section Some ‘traditional’ normative positions. As different as they might be, both in details and motivations, the accounts of disease that we previously reviewed—given by Nordenfelt, Fulford, Megone, Reznek, Cooper, and Wakefield—all count as strongly normative in our sense: all of them do explicitly contain an evaluative concept, such as disability, action failure, harm, suffering, unluckiness, or undesirability, as an explicit component of the concept of disease.^15^

Conversely, the concept of disease is weakly normative if no evaluative concept explicitly figures as a component of the definition of disease, but some value-laden concepts or judgments may intervene in the operationalization of some of such components. Kingma’s third- and fourth-level value-intrusion and Cooper’s belt-and-brace strategy immediately fit our characterization of weak normativism. Let us briefly recap why. First, these are views in which disability, action failure, harm, suffering, unluckiness, undesirability, or other similar evaluative concepts do not figure as explicit ingredients of the disease concept. Specifically, Kingma analyzes Boorse’s biostatistical theory, which is function-requiring only, and Cooper focuses on the recent consensus that harm is no longer a component of the definition of mental disorder. Second, values are only needed to operationalize the notion of dysfunction or justify such operationalization: in the fixing of thresholds between functional and dysfunctional indicators relative to a reference class, in the selection of reference classes, and in the individuation of typical environments.

We shall now argue for three claims. First, weak and strong normativism about the disease concept are philosophically grounded on different backgrounds and motivations. Second, weak and strong normativity about disease may imply different judgments about whether or not a condition is a disease. Third, the evaluative component enters into considerations with reference to two different domains (that of the population and that of the individual).

First, weak and strong normativisms about disease are philosophically substantiated by different backgrounds and motivations. As we showed in section Some ‘traditional’ normative positions, strong normativism assumes from the very beginning that disease is something that interferes with human well-being and flourishing and thus is a bad thing to have, something that we (individuals or society) negatively evaluate and dislike it being constitutively linked to some disability, action failure, harm, suffering, unluckiness or undesirability; conversely, weak normativism—as described in sections Kingma’s ‘third- and fourth-level’ strategy and Cooper’s “belt-and-brace” strategy —moves from a naturalist definition of disease, which typically defines disease in terms of dysfunction, and does not explicitly include any evaluative concept. Moreover, if strong normativism typically endorses a holistic concept of disease, weak normativism is instead compatible with the idea of disease as part-dysfunction. Finally, if strong normativism implicitly refuses the idea that medicine is a theoretical discipline, like biology or chemistry, and conceives it as a normative and prescriptive discipline, a practice, or an art, weak normativism is instead compatible with the idea that medicine, or at least its core, is to be considered a theoretical science. Backgrounds are thus quite diverse. On the side of motivations, weak normativism aims at showing that the concept of disease, even when it is defined in terms of dysfunction, conceals some evaluative components and thus is not purely factual; on the contrary, strong normativism claims that dysfunction is neither necessary nor sufficient to define what a disease is (as for most traditional normativisms) or, at least, that it is not sufficient (as for hybrid theories). In any case, the concept of disease must explicitly involve, as its components, notions such as action failure, disability, harm, and suffering, that is, notions that are intrinsically value-laden and holistic. Moreover, strong normativists would not be satisfied to say that the concept of disease is as much value-laden as many other scientific notions (see section Weak/strong normativity vis à vis with other similar distinctions for some examples), as they see it as a negative concept that ‘calls for action’, so as to restore health.

Second, weak and strong normativism may imply different judgments about whether or not a certain condition counts as a disease and, derivatively, whether or not certain individuals should be judged diseased. For instance, let us consider hypertension. According to weak normativism, deciding whether or not a certain blood pressure level (let’s say 140–90) counts as hypertension might require taking into account evaluative considerations, as Kingma and Cooper both suggest, as it would be important to balance the harm and impairment caused by the condition and the possible benefits of its treatment. Even if such considerations are grounded in practical and moral values, they are typically made at the level of population (through randomized controlled trials), not of the individual subjects, though. This means that, as treatments are easy and cheap, a blood pressure of 140–90 might be dubbed a disease (hypertension), and a subject with this blood pressure diseased, by weak normativism; conversely, as this condition generally causes no experienced harm, suffering or disability to the individual subject, it would not be negatively evaluated nor count as a disease, and the subject would not be considered diseased, by strong normativism.

A similar case involves the classification of prostate cancers. These tumors are graded through a system called Gleason score, according to their morphology and prognosis. The grading system until 2014 privileged morphology, so that cell formations that were likely to be clinically unharmful, but were nevertheless abnormal, could reach Gleason score 6, which was the threshold of clinical significance. In 2014 the classification was changed in a consensus conference based on evidence, and a new scoring system was introduced. The change was made with the aim of increasing precision of tumor stratification, but also and most importantly in order to prevent overtreatment and unnecessary psychological harm to patients. When patients were told that they had a Gleason score 6 out of 10, it implied that their prognosis was intermediate and contributed to their fear of having a more aggressive cancer. With the new system, abnormal cell formations with good prognosis at the population level are not prostate cancers anymore (Epstein et al., 2016). The prostate cancer grading change can be described as the application of a weakly normativist concept of disease, in that values (overtreatment and anxiety are bad) and goals (prevention of serious disease and death) intervene in the new system in determining the score and therefore in setting the threshold of clinical significance. Such values and goals, however, are evaluations partially based on data about survival and patients’ quality of life obtained from randomized controlled trials and other studies on prostate cancer prognosis, not on judgments on the harm, suffering, unluckiness, disability, or action failure that an individual patient may suffer. This means that a subject may be regarded as diseased by weak normativists and non diseased by strong normativists.

The above examples underlie a third important difference between weak and strong normativism. In the former case, the evaluative component of disease typically enters into considerations with reference to the population, not to the individual subject. In the latter, however, the fact that disease is negatively evaluated and disliked is determined with regard to the individual subject as a whole (or in Reznek’s case the standard subject), thus taking into account his or her experiential point of view. To put it differently, in weak normativism harm is related to the possibilities and opportunities of interventions, at the populational level, while in strong normativism harm is related to what is bad and undesirable for the individual person as a whole.

### Weak/strong normativity vis à vis with other similar distinctions

At this point it is important to address another issue, which concerns the novelty of our position. To begin, there are other authors who have already introduced the distinction between weak and strong normativism, but, as we will explain below, their distinctions do not map onto ours.

For instance, according to Boorse, strong normativism can be used to label those theories that regard diseases as “pure evaluations without descriptive meaning”, and are thus purely social constructivist, while weak normativism allows “a descriptive as well as a normative component” (Boorse, 1975, p. 51). Both of Boorse’s kinds of normativism, however, would be labeled as strong according to our distinction. Also Khushf (Khushf, 2007, pp. 24–25) makes a distinction between strong and weak normativism. On the one hand, there are purely social constructivists in Boorse’s sense and also skeptics about the disease/non-disease demarcation project, who think that the very distinction between facts and values should be abandoned; on the other hand, as for Boorse, there are those who consider values as a necessary component of the concept of disease but allow for descriptive and factual considerations, too. Again, both these kinds of normativism would be labeled strong according to our distinction.

As we discussed above, Kingma’s and Cooper’s frameworks somehow map onto our distinction. To begin with, Kingma’s second-level normativism can be equated with our strong normativism and her third- and fourth-level normativisms to our weak normativism (see sections Kingma’s ‘third- and fourth-level’ strategy and Weak and strong normativity). Cooper’s case is slightly more complicated. As we argued above, we regard her belt-and-brace strategy as a kind of weak normativism, as values do not explicitly appear in the disease definition but play a role in the operationalization of some component of this definition (see sections Cooper’s “belt-and-brace” strategy and Weak and strong normativity). However, Cooper seems to conflate weak and strong normativism under the same ‘normativist’ label, to be opposed to naturalism about disease, where “the naturalists [are seen] as claiming that disorder is at least no more value-laden than other biological concepts, such as life or cell” (Cooper, 2020, p. 144). Her framework thus appears to be less nuanced than ours.

In the rest of this section, we will argue that many scientific concepts can be considered weakly normative in our sense: they do not have any evaluative component as an explicit part of their definition, but evaluative considerations play a role in the operationalization of some of such components.^16^ This is important for two reasons. With regard to Cooper’s framework, it means that her belt-and-brace strategy may be seen in the opposite way to what she does: not as a kind of normativism but as a kind of naturalism (according to her own terminology). More generally, with regard to the normativist-naturalist debate about disease, it means that weak normativism would be better understood as a form of value-conscious naturalism.

Let us start with a medical concept, the underlying cause of death, as defined and regulated by the World Health Organization—for a more extensive discussion, see (Amoretti & Lalumera, 2021). According to the WHO’s definition, the underlying cause of that is “the disease or injury that initiated the sequence of morbid events that led directly to death” (World Health Organization, 1979, p. 6).^17^ Apparently, it seems that this concept is value free and could be determined simply by looking at biomedical evidence, that is, considering pure data and facts. However, the WHO’s rules explicitly require that the choice of the underlying cause of death must be done with the primary objectives of *prevention and treatment* in mind (World Health Organization, 1979, 2018), that is, taking into account non-epistemic factors, such as values and practical goals—values and practical goals that, in particular circumstances, might even override the biomedical evidence. For example, HIV/AIDS or COVID-19 should be classified as the underlying cause of death, even when other death-causing conditions are equally compatible with the available evidence of the patient’s assessment and history, because this is advantageous from the point of view of infection control. The scientific concept of underlying cause of death is therefore not explicitly value-laden, but values intervene in the rules for its application, as these rules are context-sensitive and value-laden. Thus, the concept of underlying cause of death is weakly normative according to our definition.

Another slightly different example is the confidence interval. This is a statistical concept that comes from epidemiology, where conclusions about the effect of an exposure on an outcome in a population are drawn from data taken from a sample, and it is an essential component of quantitative research. Here, the statistical concept of confidence interval can be seen as expressing the range of false positives and false negatives, that is, intuitively, how much a study can be mistaken in finding a correlation when in fact there is none (false positives) and not finding a correlation when in fact there is (false negatives). When false negatives are evaluated as more dangerous than false positives—for example, when there is a relevant public health risk—a larger confidence interval is tolerated and can even be intentionally chosen. This can be the case, for example, of assessing the correlation between heavy metals in drinking water and severe headaches. Differently, smaller confidence intervals are chosen when false positives are to be avoided, as when testing whether, say, a vitamin C supplement is protective against the common cold. The threshold of acceptability of a confidence interval is therefore context-dependent and value-laden (Carolan, 2006; Douglas, 2000); it is weakly normative in that no non-epistemic value is a component of its definition, but non-epistemic values can fix the conditions for its application.

A third example of a scientific concept with a surprising but arguably innocent value-intrusion is provided by Schwartz in his discussion of the boundaries of the concept of disease:

even classic natural kind terms may have vague boundaries that appear to have been set by stipulation or historical contingency. The concept of water includes H_2_O molecules. That counts as a type of water (‘heavy water’), but it seems possible that when isotopes were discovered, ^2^H_2_O could have been excluded from the concept ‘water’. (Schwartz, 2017, p. 497).

Heavy water contains deuterium rather than the common hydrogen isotope protium, and has different effects than normal water on mammals, and on humans in particular. There is no epistemic reason, however, for choosing protium over deuterium—the reasons have to do with how they relate to our explanatory interests. Therefore, as the extension of the natural and scientific concept of water is restricted so as to exclude heavy water, this counts as a case of value-intrusion and thus of weak normativism.

The three examples above are merely illustrative of a vast literature aimed at showing how the scientific enterprise is not, nor can it be, completely free from ethical, political, and social values, that is, on non-epistemic values. Specifically, our examples about the concepts of underlying cause of death and water may be easily related to the discussion on how what counts as evidence is dependent on non-epistemic values (Longino, 1979, 1990). Moreover, our example about the confidence interval is clearly connected to the recent discussion on inductive risk, which shows that what counts as sufficient evidence in the evaluation of scientific hypotheses as well as many other aspects of the scientific practice, such as the characterization of data, cannot avoid the intrusion of non-epistemic values in weighing the consequences of potential error (Rudner, 1953; Douglas, 2000, 2009; Elliott and Richards, 2017). On a very general level, one way to categorize these arguments is to see them as contributions to redefining the notion of objectivity of science and scientific concepts. Having discarded the idea of objectivity as absence of values, or as “a view from nowhere” (absence of perspective or standpoint), many philosophers of science are now developing accounts of scientific objectivity, which include notions such as trustworthiness and pluralistic debate.^18^ More locally, with respect to our point, the above arguments are relevant as they show that if the concept of disease is normative only in our weak sense, then it is no more value-laden than many other scientific concepts, such as those of underlying cause of death, confidence interval, water, and many others. As we anticipated, this has two important consequences.

First, with regard to Cooper’s framework and terminology, we argue that her belt-and-brace strategy may be better seen as a form of naturalism rather than a form of normativism. In fact, following the belt-and-brace strategy, the concept of disease would be only weakly normative and thus no more value laden than other scientific concepts. Accordingly, as Cooper sees naturalists as claiming that disorder is no more value-laden than other scientific concepts, her belt-and-brace strategy should be described as a form of naturalism. Thus, not only does her conceptual framework appear somewhat contradictory, but it would still include only two main categories, naturalism and normativism, instead of three (as Kingma’s framework and ours—see section Kingma’s ‘third- and fourth-level’ strategy above and section Towards a better framework for the normativist-naturalist debate about disease below).

Second, we argued that *if* weak normativism holds, *then* the concept of disease would not be more value-laden than many other scientific concepts, or *any* other scientific concept. If this characteristic of weak normativity is explicit in our account, it is not immediately clear either in Kingma’s or in Cooper’s distinction. Moreover, both their frameworks seem to imply that a naturalist position about the concept of disease cannot support any role for values, which commits all naturalisms to a particularly strong claim. To improve conceptual clarity, we thus suggest that weak normativism would be better understood as a form naturalism about disease, specifically a value-conscious naturalism, as it is compatible with the fact that many other scientific concepts (or perhaps *any* other scientific concept) depend(s) on considerations that are not purely factual. In the following section we will expand this idea and wrap up our argument.

### Towards a better framework for the normativist-naturalist debate about disease

For the reasons discussed in the previous section (section Weak/strong normativity vis à vis with other similar distinctions) and given the great dissimilarities between strong and weak normativity (section Weak and strong normativity), we propose not to use the same label of ‘normativism’ to refer to both sets of positions. Accordingly, we suggest that the term ‘normativism’ should be used to refer solely to what we dubbed strong normativism, that is, to traditional normativist positions, while the expression ‘value-conscious naturalism’ would be preferable to indicate weak normativism. We believe that this choice would not only improve conceptual clarity but also contribute to clarifying the normativist-naturalist debate about disease, where the term ‘normativism’ is often used in slightly different ways, with non-overlapping meanings. More precisely, in our view the conceptual framework about the disease concept should be reframed as follows.

First, the label ‘naturalism’ (or ‘strong naturalism’) should be used to refer to those who think that the concept of disease is purely factual and value-free. In this sense, values are not only to be excluded from the explicit definition of the concept of disease, but they do not have to play any role in the operationalization of the components of such definition, too.^19^ Second, the label ‘normativism’ should be used to refer to those who think that the concept of disease is constitutively ‘value-requiring’ (and thus more value-laden than other scientific concepts) in that it must be explicitly defined using value-laden concepts, such as disability, action failure, harm, suffering, unluckiness, or undesirability. In light of the above discussion, traditional normativist positions would continue to be considered normativist in this sense (section Some ‘traditional’ normative positions). Third, the label ‘value-conscious naturalism’ (instead of ‘weak normativism’) should be used to refer to those who think that the concept of disease is not to be explicitly defined in terms of value-laden concepts but recognize that an evaluative component can still be present, as it is present in many other scientific concepts. Specifically, the evaluative component may play a role in the operationalization of some elements of the definition of the concept of disease and thus ‘value intrusion’ can be better explained in terms of interest-ladenness or contextual dependence.

Even if value-conscious naturalism corresponds to what we previously dubbed ‘weak normativism’ and to Kingma’s third- and fourth-level normativism, too,^20^ we believe that it would be better conceived as a form of naturalism rather than as a form of normativism. To begin, this category strongly differs from traditional nomativist positions, as we explained in section Weak and strong normativity: it has different backgrounds and motivations, leads to different judgements on what counts as a disease, and considers the evaluative component at different levels (populational vs. individual). Moreover, this middle ground position is compatible with the idea, which is dear to naturalists, that medicine, or at least its core, is to be considered a ‘theoretical’ science (Boorse, 1997), as values in medicine would play the same role that they play in any other scientific endeavor. Traditional normativists, on the other hand, tend to see the concept of disease as fundamentally different from other scientific concepts and would not be satisfied to say that it is as much value-laden as many other scientific notions: the concept of disease is in fact explicitly defined as a negative concept that ‘calls for action’, so as to restore health (section Some ‘traditional’ normative positions). Sometimes traditional normativists also prefer to regard medicine as an art or a technique rather than a (theoretical and empirical) science as it “is an activity whose essence appears to lie in the clinical event, which demands that scientific and other knowledge be particularized in the lived reality, of a particular human, for the purpose of attaining health or curing illness, through the direct manipulation of the body, and in a value-laden decision matrix” (Pellegrino & Thomasma, 1981, p. 26). Moreover, value-conscious naturalism is compatible with some of the attempts to reconcile the value-ladenness of science with its objectivity and rationality, thus preserving the objectivity of the disease concept, whether traditional normativism may easily incur in relativistic outcomes about the disease concept–as, for instance, it is explicitly recognized by Cooper, who claims that “one and the same condition can be pathological for one person but not for another” (2002, p. 274).

Value-conscious naturalism is not only more theoretically accurate as a classification of positions in the current philosophical debate; we also think that it is descriptively adequate of actual tendencies in medical research and practice. Assessment of outputs and endpoints of experimental studies, such as randomized controlled trials, is increasingly value-laden, and recognized as being so by leading practitioners (Porter, 2010; Porter et al., 2016). In particular, methodologists recommend that outcomes, for example the effect of a new drug or of an intervention, are patient-relevant, and not just robust or merely statistically significant.^21^ Moreover, measures such as ‘QALY’ (quality-adjusted life years) and ‘DALY’ (disability-adjusted life years), and concepts such as ‘appropriateness’, which incorporate economic and pragmatic values,^22^ are central to the design and assessment of research (Alexandrova, 2017). The presence of this kind of non-epistemic values in medical research has the ultimate aim of connecting research with public health policies, and ultimately with patients’ good.

A possible objection to our overall project in this paper can be clearly put as follows: Why does it matter whether the middle ground position that Kingma, Cooper and ourselves are all trying to describe gets called a form of naturalism rather than a form of normativism? Our answer is that this rebranding or relabeling is not neutral, as it brings with it some positive consequences. First, labeling it as ‘naturalism’ may help to bridge the gap between the debate in the philosophy of medicine about the concept of disease and the debate in the general philosophy of science about the value-free ideal. One of the major insights of recent general philosophy of science is that values in science are not a despicable intrusion but can be incorporated into a broader notion of objectivity. The normativist-naturalist opposition in the philosophy of medicine somehow thrived in isolation from this insight, and value-conscious naturalism may help break this isolation. On a very general level, it is often positive when two philosophical debates are shown to merge. Second, our rebranding can be seen as a kind of conceptual engineering or conceptual amelioration (see, e.g. Burgess et al., 2020). We think that a concept of disease that proves to be both unproblematically scientific and value-laden as many other scientific concepts, such as the one we advocate, could help integrate the social and ethical dimensions of disease into medical research and practice. It could help weaken the polarization between ‘hard’ biological models of disease and biopsychosocial models, recognizing a common core to both.

To sum up, we first addressed what we take to be the most representative normative positions in the debate on the concept of disease in order to find out what they have in common: all of them are ‘value requiring’, in that values and norms figure as necessary components of the concept of disease, and holistic, as the concept of disease is taken to refer to the whole person. Then, we reviewed Kingma’s ‘third- and fourth-level’ and Cooper’s ‘belt-and-brace’ strategies to reframe normativism. In the light of this, we introduced our distinction between weak and strong normativity and illustrated how it differs from similar ways to map the different varieties of normativism. Finally, we proposed to use the term ‘normativism’ to refer solely to what we dubbed strong normativism, that is, to traditional normativist positions, and the expression ‘value-conscious naturalism’ to indicate weak normativism. This would not only improve conceptual clarity but also describe current tendencies in medical research and practice.

## Data Availability

Not applicable.

## Footnotes

1 The two most common rival accounts of function are Boorse’s forward-looking or causal contribution account, and Wakefield’s backward-looking or etiological account of function (Boorse, 1975, 1976, 1977; Wakefield, 1992, 1995, 1999). The debate on the notion of function is now huge and its assessment is not among the aims of our present discussion.

2 Broadbent (2019) observes that, from the perspective of the contemporary meta-ethical and metaphysical literature, it is misleading to equate ‘natural’ with ‘value-free’, and ‘value-laden’ with ‘subjective’, and thus proposes a different taxonomy of positions about the concepts of health and disease, also involving the judgment-dependence dimension. We do not address it further.

3 We are well aware that other relevant normativist theories have been advocated but we believe that the positions we review here are sufficient to exemplify what traditional normativists mean when they claim that the concept of disease is intrinsically value-laden. It should also be noted that, in their analyses, some normativists do not consider the notion of disease, but rather those of ‘malady’ (Nordenfelt), ‘illness’ (Megone), or ‘disorder’ (Wakefield); however, as we have already clarified, for the aims of this paper all these concepts can be equated to that of disease, which is used as an umbrella term for all pathological conditions.

4 More precisely, Nordenfelt distinguishes between first-order and second-order ability, and identifies being healthy with having the *second-order* ability to achieve a minimal degree of happiness. First order abilities are, for example, the ability to speak a language, walk, or read, while a second-order ability is the ability to follow a training or learning path at the end of which one will be able to have a first-order ability.

5 See, e.g., (Hanna, 2016) for a counterfactual comparative account of harm.

6 There is consensus on the claim that naturalism is generally a revisionary position. See, e.g., (Murphy, 2020; Schwartz, 2017).

7 For further discussions, see also (Hausman, 2012, 2014; Schwartz, 2017).

8 Oncologists recently debated the classification of prostate cancer; see, e.g., (Epstein et al., 2016).

9 Boorse writes that “Local part dysfunctions need not have any gross effects on disability or deformity or distress. […] Liver cells, to be normal, must perform a host of metabolic functions because that is what liver cells collectively contribute to survival and reproduction. But a large number of liver cells can be pathological without clinically detectable effects or appreciable risk of such effects” (Boorse, 1987, pp. 371–372). His theory clearly distinguishes between disease as dysfunction, and conditions that calls for medical treatment (Boorse, 1997) See also (Kincaid, 2008) for a different approach to the problem of demarcating cancers.

10 In most of her works Cooper focuses on the concept of disorder as it is used in the debates between psychiatrists, psychologists, mental health workers, and patients or lobby groups—see, e.g., (Cooper, 2015a). It would be neither correct to locate her project on Kingma’s first level—that of ordinary use description—nor on the second level—that of “conceptually clean” accounts. What interests us, however, is value-intrusion normativity that both philosophers individuate, and which can be independently characterized without Kingma’s conceptual apparatus of levels.

11 As we said in the opening, we aim our discussion at the umbrella concept of disease, intended as a superordinate concept. It might be the case that Cooper would not accept her argument to be extended to the concept of disease, either, but we are not interested in an exegesis of her paper. We therefore make explicit that the interpretation of Cooper’s “belt-and-brace” strategy as being about the concept of disease is only ours.

12 We acknowledge that (typically) normativists’ arguments may have more grip in the case of psychiatry.

13 Arguably, this is a conceptual defect of the manual. In a recent paper Amoretti and Lalumera (2019) suggested that harm should be explicitly ‘unpacked’ for every DSM diagnostic category, to specify whether it is harm for the subject, his or her close acquaintances, or the society, and who can assess it.

14 Cooper individuates the relational model of disability as one of the forces that produced the conceptual shift of mental disorder towards naturalism.

15 If disease is strongly normative, then it is probably a thick concept in the traditional sense of having both a descriptive and an evaluative component—see, e.g., (Kirchin, 2013).

16 Of course, if the concept of disease has an evaluative component as an explicit part of its definition, as strong normativism claims, it clearly differs from most other scientific concepts, which lack such an evaluative component.

17 To be precise, the WHO’s definition of the underlying cause of death comprises another disjunct, that is “the circumstances of the accident or violent act that produced the fatal injury”; however, it is not relevant here.

18 See John 2021 for an updated review and discussion of “new-wave” objectivity.

19 Strong naturalism is a bold position that, for instance, can be ascribed to Boorse (1977) and Schwartz (2007b).

20 To repeat, we do not mention Cooper’s framework here as it would be less nuanced than Kingma’s and ours, as it only has two categories instead of three (section Weak/strong normativity vis à vis with other similar distinctions).

21 Jeremy Howick (2011) also makes a case for patient-relevant outcomes in medical research.

22 For a more thorough discussion of values in DALY see (Solberg et al., 2020).
